# 2,25-Dioxo-27,28-diphenyl-30-oxa-29-thia-3,10,17,24-tetra­aza­penta­cyclo­[24.2.1.1^12,15^.0^4,9^.0^18,23^]triaconta-5,7,9(4),10,12,14,16,18,20,22,26,28-dodeca­ene chloro­form disolvate

**DOI:** 10.1107/S1600536810006173

**Published:** 2010-02-20

**Authors:** Rizvan K. Askerov, Vladimir V. Roznyatovsky, Evgeny A. Katayev, Abel M. Maharramov, Victor N. Khrustalev

**Affiliations:** a Baku State University, Z. Khalilov St 23, Baku, AZ-1148, Azerbaijan; b Chemistry Department, M.V. Lomonosov Moscow State University, Leninskie gory 1/3, Moscow, 119991, Russian Federation; c A.N. Nesmeyanov Institute of Organoelement Compounds, Russian Academy of Sciences, Vavilov St 28, B-334, Moscow 119991, Russian Federation

## Abstract

The macrocycle of the title compound, C_36_H_24_N_4_O_3_S·2CHCl_3_, contains a rigid framework with the nitro­gen and oxygen heteroatoms pointing in towards the center of the macrocyclic cavity. The macrocycle is essentially planar (r.m.s. deviation = 0.027 Å) except for the thio­phene ring. The dihedral angle between the thio­phene ring plane and the mean plane of the central macrocyclic core including all atoms except sulfur is 21.6 (1)°. Four intra­molecular hydrogen bonds occur: two are between the amide hydrogen atoms and the Schiff base nitro­gen atoms, while the others are between the amide hydrogen atoms and the sulfur atom of the thio­phene. The two solvate chloro­form mol­ecules are bound to the carbonyl oxygen atoms of the ligand by weak C—H⋯O hydrogen bonding. In addition, the structure reveals inter­molecular Cl⋯Cl close contacts [3.308 (2), 3.404 (2) and 3.280 (2) Å] between the chloro­form solvate mol­ecules. In the crystal, the macrocycles form layers parallel to (101), with an inter­layer distance of 3.362 (3) Å. This short distance is determined by the stacking inter­actions between the amide carbonyl and imine fragments of neighboring ligands.

## Related literature

For general background to biological anion–receptor inter­actions, see: Caltagirone & Gale (2009 ▶). For the synthesis of synthetic anion receptors, see: Aydogan *et al.* (2008 ▶). For related compounds, see: Sessler *et al.* (2005*a* ▶,*b* ▶).
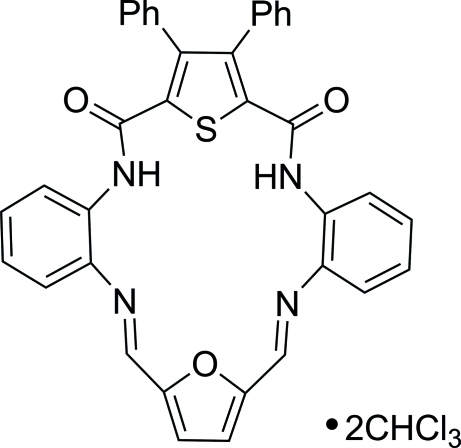


## Experimental

### 

#### Crystal data

C_36_H_24_N_4_O_3_S·2CHCl_3_
*M* *_r_* = 831.40
Monoclinic, 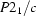

*a* = 13.0957 (15) Å
*b* = 31.854 (3) Å
*c* = 8.7368 (9) Å
β = 98.857 (3)°
*V* = 3601.1 (7) Å^3^
*Z* = 4
Mo *K*α radiation
μ = 0.58 mm^−1^
*T* = 120 K
0.30 × 0.24 × 0.21 mm

#### Data collection

Bruker SMART 1K CCD diffractometer
Absorption correction: multi-scan (*SADABS*; Sheldrick, 1998 ▶) *T*_min_ = 0.845, *T*_max_ = 0.888
29858 measured reflections
7802 independent reflections
4582 reflections with *I* > 2σ(*I*)
*R*_int_ = 0.061

#### Refinement

*R*[*F* ^2^ > 2σ(*F* ^2^)] = 0.050
*wR*(*F* ^2^) = 0.108
*S* = 1.01
7802 reflections
469 parameters
H-atom parameters constrained
Δρ_max_ = 0.42 e Å^−3^
Δρ_min_ = −0.29 e Å^−3^

### 

Data collection: *SMART* (Bruker, 1998 ▶); cell refinement: *SAINT-Plus* (Bruker, 1998 ▶); data reduction: *SAINT-Plus*; program(s) used to solve structure: *SHELXS97* (Sheldrick, 2008 ▶); program(s) used to refine structure: *SHELXL97* (Sheldrick, 2008 ▶); molecular graphics: *SHELXTL* (Sheldrick, 2008 ▶); software used to prepare material for publication: *SHELXTL*.

## Supplementary Material

Crystal structure: contains datablocks global, I. DOI: 10.1107/S1600536810006173/rk2192sup1.cif

Structure factors: contains datablocks I. DOI: 10.1107/S1600536810006173/rk2192Isup2.hkl

Additional supplementary materials:  crystallographic information; 3D view; checkCIF report

## Figures and Tables

**Table 1:** Hydrogen-bond geometry (Å, °)

*D*—H⋯*A*	*D*—H	H⋯*A*	*D*⋯*A*	*D*—H⋯*A*
N1—H1*N*⋯N2	0.90	2.13	2.622 (3)	114
N1—H1*N*⋯S1	0.90	2.48	2.970 (3)	115
N4—H4*N*⋯N3	0.90	2.13	2.620 (3)	114
N4—H4*N*⋯S1	0.90	2.53	2.986 (3)	112
C32—H32⋯Cl6^i^	0.95	2.69	3.461 (3)	139
C37—H37⋯O1	1.00	2.34	3.149 (3)	137
C38—H38⋯O3	1.00	2.46	3.205 (3)	131

Symmetry code: (i) 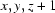.

**Fig. 1.**
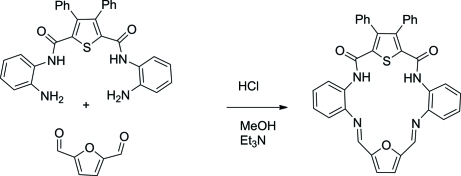
Synthesis of the macrocyclic ligand I.

**Fig. 2.**
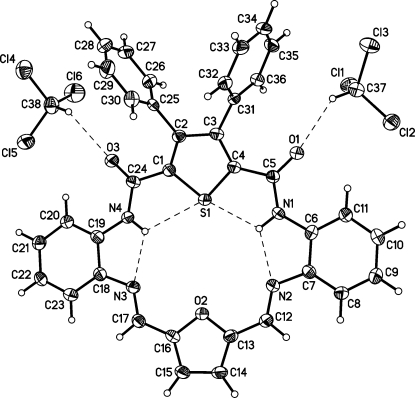
Molecular structure of I with the atom numbering scheme. Displacement ellipsoids are shown at the 50% probability level. H atoms are presented as a small spheres of arbitrary radius. Dashed lines indicate the hydrogen bonds.

**Fig. 3.**
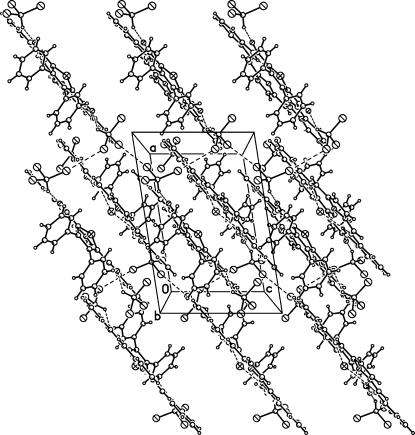
Crystal packing of I. Dashed lines indicate the hydrogen bonding and Cl···Cl interactions.

## supplementary crystallographic information

### Comment

The ubiquity of anions in nature makes an understanding of biological anion–receptor interactions a topic of considerable current interest (Caltagirone & Gale, 2009). It is also inspiring the synthesis of synthetic anion receptors, systems whose potential utility could span the full spectrum of applications from separations and waste remediation to biomedical analysis and therapy (Aydogan *et al.*, 2008). We are particularly interested in the design of rigid macrocyclic hosts for anions and use for this purpose aromatics linked by amide or imine bonds. These bonds and pyrrole rings serve as efficient coordination site for anions functioning by means of hydrogen bonds. In our previous works, it has been shown that rigid scaffold of a receptor results in a higher selectivity that the one with flexible skeleton (Sessler *et al.*, 2005a,b). In this work, we present the new receptor bearing furan and thiophen-2,5-dicarboxamide units in one macrocycle.

The target receptor was synthesized according to the method of template synthesis using chloride anion as a template. The dialdehyde (2,5–diformylfuran) and diamine (*N,N*'-bis(2-aminophenyl)-3,4-diphenylthiophen-2,5-dicarboxamide) were condensed in the presence of hydrochloric acid affording hydrochloric acid salt of the macrocyclic receptor I. The HCl that was subsequently neutralized by triethylamine to give free base ligand I (Fig. 1). The single crystals of I suitable for X–ray diffraction analysis were obtained by slow crystallization from chloroform–methanol mixture.

The title compound I crystallizes as a solvate with two chloroform molecules, *i. e.*, C_36_H_24_N_4_O_3_S^.^2CHCl_3_. The macrocycle I contains a rigid framework with the N1, N2, O2, N3 and N4 heteroatoms pointing in toward the center of the macrocyclic cavity (Fig. 2). It is practically planar excepting for the thiophene ring. By the intermolecular C—H···O hydrogen bond (Table 1), the phenyl group at the C3 carbon atom of the thiophene forces this ring to deviate from the plane of the central macrocyclic core passed through the C1/C4/C5/N1/C6/C7/N2/C12/C13/O2/C16/C17/N3/C18/C19/N4/C24 atoms (the dihedral angle is 21.6 (1)°. There are four internal hydrogen bonds in I. Two are between the amide NH protons and the Schiff base nitrogen atoms, while the other are between the amide NH protons and the sulfur atom of the thiophene (Table 1). The two solvate chloroform molecules are bound to the carbonyl oxygen atoms of the ligand by weak C—H···O hydrogen bonding (Table 1). In addition to these effects, the structure reveals the intermolecular Cl···Cl attractive interactions between the chloroform solvate molecules (Cl1···Cl3^ii^, Cl1···Cl4^iii^ and Cl2···Cl5^iv^ distances are 3.308 (2)Å, 3.404 (2)Å and 3.280 (2)Å, respectively). In the crystal, the macrocycles I form the layers parallel to (101), with the interlayer distance of 3.362 (3)Å (Fig. 3). This short distance is determined by the stacking interactions between amide carbonyl and imine fragments of neighboring ligands. Symmetry codes: (ii) *x*, -*y*+1/2, *z*-1/2; (iii) *x*-1, *y*, *z*; (iv) *x*-1, *y*, *z*+1.

### Experimental

Concentrated hydrochloric acid (21.5 µl, 0.18 mmol) was added to a mixture of 2,5-diformylfuran (13 mg, 0.1 mmol) and *N,N*'-bis(2-aminophenyl)-3,4-diphenylthiophen-2,5-dicarboxamide (51 mg, 0.1 mmol) in 20 ml dry *Me*OH. The colourless clear solution was stirred for overnight at 296 K. The precipitate formed was filtrated off and suspended in 1.5 ml dry dichloromethane. Then triethylamine (25.3 µl, 0.18 mmol) was added to the suspension affording yellow clear solution, which was passed through a plug of silica gel. Evaporation of the solvent yielded 44 mg (73%) of product I. M.p. > 623 K (decomp.). Found (%): C, 72.87; H, 4.08; N, 9.43. Calcd. for C_36_H_24_N_4_O_3_S (%): C, 72.96; H, 4.08; N, 9.45. ^1^H NMR (CDCl_3_, 293 K): \d = 7.05–7.24 (m, 16H), 7.36 (d, 2H), 8.50 (d, 2H), 8.52 (s, 2H), 10.38 (s, 2H). ^13^C NMR (CDCl_3_, 293 K): \d = 114.67, 119.41, 120.56, 123.68, 127.37, 127.54, 129.43, 130.12, 132.25, 134.41, 135.43, 135.82, 142.66, 148.52, 154.21, 157.99. Mass spectrum (MALDI–TOF), m/z (I_r_, %): 593.10 [*M*+*H*]^+^.

### Refinement

The hydrogen atoms were placed in calculated positions with N—H = 0.88Å and C—H = 0.95–1.00Å and refined in the riding model with fixed isotropic displacement parameters: *U*_iso_(H) = 1.5*U*_eq_(C) for CH_3_-groups and *U*_iso_(H) = 1.2*U*_eq_(N or C) for the other groups.

### Crystal data

**Table:**

C_36_H_24_N_4_O_3_S·2CHCl_3_	*F*(000) = 1696
*M**_r_* = 831.40	*D*_x_ = 1.533 Mg m^−^^3^
Monoclinic, *P*2_1_/*c*	Mo *K*α radiation, λ = 0.71073 Å
Hall symbol: -P 2ybc	Cell parameters from 5725 reflections
*a* = 13.0957 (15) Å	θ = 2.5–26.6°
*b* = 31.854 (3) Å	µ = 0.58 mm^−^^1^
*c* = 8.7368 (9) Å	*T* = 120 K
β = 98.857 (3)°	Prism, dark–orange
*V* = 3601.1 (7) Å^3^	0.30 × 0.24 × 0.21 mm
*Z* = 4	

### Data collection

**Table:**

Bruker SMART 1K CCD diffractometer	7802 independent reflections
Radiation source: fine–focus sealed tube	4582 reflections with *I* > 2σ(*I*)
graphite	*R*_int_ = 0.061
φ– and ω–scans	θ_max_ = 27.0°, θ_min_ = 1.7°
Absorption correction: multi-scan (*SADABS*; Sheldrick, 1998)	*h* = −16→16
*T*_min_ = 0.845, *T*_max_ = 0.888	*k* = −40→40
29858 measured reflections	*l* = −11→11

### Refinement

**Table:**

Refinement on *F*^2^	Primary atom site location: structure-invariant direct methods
Least-squares matrix: full	Secondary atom site location: difference Fourier map
*R*[*F*^2^ > 2σ(*F*^2^)] = 0.050	Hydrogen site location: inferred from neighbouring sites
*wR*(*F*^2^) = 0.108	H-atom parameters constrained
*S* = 1.00	*w* = 1/[σ^2^(*F*_o_^2^) + (0.045*P*)^2^] where *P* = (*F*_o_^2^ + 2*F*_c_^2^)/3
7802 reflections	(Δ/σ)_max_ = 0.001
469 parameters	Δρ_max_ = 0.42 e Å^−^^3^
0 restraints	Δρ_min_ = −0.29 e Å^−^^3^

### Special details

**Table:**

Geometry. All s.u.'s (except the s.u. in the dihedral angle between two l.s. planes) are estimated using the full covariance matrix. The cell s.u.'s are taken into account individually in the estimation of s.u.'s in distances, angles and torsion angles; correlations between s.u.'s in cell parameters are only used when they are defined by crystal symmetry. An approximate (isotropic) treatment of cell s.u.'s is used for estimating s.u.'s involving l.s. planes.
Refinement. Refinement of *F*^2^ against ALL reflections. The weighted *R*–factor wR and goodness of fit *S* are based on *F*^2^, conventional *R*–factors *R* are based on *F*, with *F* set to zero for negative *F*^2^. The threshold expression of *F*^2^ > σ(*F*^2^) is used only for calculating *R*–factors(gt) etc. and is not relevant to the choice of reflections for refinement. *R*–factors based on *F*^2^ are statistically about twice as large as those based on *F*, and *R*–factors based on ALL data will be even larger.

### Fractional atomic coordinates and isotropic or equivalent isotropic displacement parameters (Å^2^)

**Table:**

	*x*	*y*	*z*	*U*_iso_*/*U*_eq_	
S1	0.57191 (5)	0.45496 (2)	0.59993 (9)	0.02602 (18)	
O1	0.38015 (15)	0.39570 (6)	0.8264 (2)	0.0325 (5)	
O2	0.58881 (14)	0.58584 (5)	0.6945 (2)	0.0255 (4)	
O3	0.80044 (15)	0.41512 (6)	0.4006 (2)	0.0338 (5)	
N1	0.42143 (17)	0.46535 (7)	0.8191 (3)	0.0241 (5)	
H1N	0.4641	0.4840	0.7846	0.029*	
N2	0.43040 (17)	0.54752 (7)	0.8331 (3)	0.0255 (5)	
N3	0.74814 (18)	0.56185 (7)	0.5267 (3)	0.0265 (6)	
N4	0.76316 (17)	0.48108 (7)	0.4781 (3)	0.0258 (6)	
H4N	0.7254	0.4959	0.5370	0.031*	
C1	0.6697 (2)	0.42298 (8)	0.5574 (3)	0.0248 (7)	
C2	0.6625 (2)	0.38325 (8)	0.6169 (3)	0.0238 (6)	
C3	0.5764 (2)	0.37870 (8)	0.6978 (3)	0.0229 (6)	
C4	0.5207 (2)	0.41545 (8)	0.6990 (3)	0.0228 (6)	
C5	0.4332 (2)	0.42398 (8)	0.7865 (3)	0.0253 (7)	
C6	0.3511 (2)	0.48458 (9)	0.9041 (3)	0.0260 (7)	
C7	0.3535 (2)	0.52889 (8)	0.9089 (3)	0.0245 (6)	
C8	0.2852 (2)	0.54974 (9)	0.9897 (3)	0.0286 (7)	
H8	0.2848	0.5796	0.9918	0.034*	
C9	0.2181 (2)	0.52777 (9)	1.0667 (3)	0.0326 (7)	
H9A	0.1718	0.5424	1.1214	0.039*	
C10	0.2181 (2)	0.48450 (9)	1.0643 (4)	0.0341 (8)	
H10	0.1732	0.4695	1.1203	0.041*	
C11	0.2827 (2)	0.46250 (9)	0.9815 (3)	0.0284 (7)	
H11	0.2803	0.4327	0.9775	0.034*	
C12	0.4479 (2)	0.58709 (9)	0.8440 (3)	0.0279 (7)	
H12	0.4060	0.6035	0.9006	0.033*	
C13	0.5266 (2)	0.60808 (8)	0.7761 (3)	0.0268 (7)	
C14	0.5540 (2)	0.64955 (9)	0.7810 (3)	0.0314 (7)	
H14	0.5227	0.6715	0.8308	0.038*	
C15	0.6373 (2)	0.65331 (9)	0.6985 (3)	0.0300 (7)	
H15	0.6730	0.6784	0.6810	0.036*	
C16	0.6570 (2)	0.61423 (8)	0.6485 (3)	0.0255 (7)	
C17	0.7350 (2)	0.60029 (9)	0.5601 (3)	0.0271 (7)	
H17	0.7788	0.6207	0.5250	0.033*	
C18	0.8263 (2)	0.54995 (9)	0.4407 (3)	0.0253 (7)	
C19	0.8333 (2)	0.50648 (9)	0.4126 (3)	0.0269 (7)	
C20	0.9060 (2)	0.49109 (9)	0.3278 (3)	0.0293 (7)	
H20	0.9100	0.4618	0.3091	0.035*	
C21	0.9727 (2)	0.51832 (9)	0.2706 (4)	0.0348 (8)	
H21	1.0225	0.5077	0.2122	0.042*	
C22	0.9675 (2)	0.56104 (9)	0.2977 (4)	0.0339 (8)	
H22	1.0141	0.5795	0.2583	0.041*	
C23	0.8952 (2)	0.57689 (9)	0.3816 (4)	0.0320 (7)	
H23	0.8921	0.6063	0.3993	0.038*	
C24	0.7509 (2)	0.43888 (9)	0.4706 (3)	0.0260 (7)	
C25	0.7390 (2)	0.34909 (8)	0.6047 (3)	0.0258 (7)	
C26	0.7238 (2)	0.31965 (9)	0.4887 (4)	0.0356 (8)	
H26	0.6637	0.3210	0.4125	0.043*	
C27	0.7960 (3)	0.28761 (9)	0.4816 (4)	0.0438 (9)	
H27	0.7851	0.2674	0.4007	0.053*	
C28	0.8824 (3)	0.28547 (10)	0.5916 (4)	0.0451 (9)	
H28	0.9313	0.2636	0.5880	0.054*	
C29	0.8980 (3)	0.31500 (11)	0.7067 (4)	0.0463 (9)	
H29	0.9583	0.3136	0.7825	0.056*	
C30	0.8277 (2)	0.34644 (9)	0.7135 (4)	0.0353 (8)	
H30	0.8397	0.3667	0.7940	0.042*	
C31	0.5545 (2)	0.33855 (8)	0.7748 (3)	0.0232 (6)	
C32	0.6213 (2)	0.32491 (8)	0.9032 (3)	0.0274 (7)	
H32	0.6791	0.3417	0.9442	0.033*	
C33	0.6045 (2)	0.28673 (9)	0.9726 (4)	0.0330 (7)	
H33	0.6496	0.2780	1.0628	0.040*	
C34	0.5235 (2)	0.26168 (9)	0.9119 (4)	0.0330 (8)	
H34	0.5131	0.2354	0.9584	0.040*	
C35	0.4571 (2)	0.27476 (9)	0.7830 (4)	0.0311 (7)	
H35	0.4010	0.2573	0.7404	0.037*	
C36	0.4715 (2)	0.31338 (8)	0.7147 (3)	0.0250 (6)	
H36	0.4246	0.3225	0.6271	0.030*	
Cl1	0.18557 (6)	0.31001 (2)	0.69064 (9)	0.0365 (2)	
Cl2	0.15795 (6)	0.35523 (2)	0.96738 (9)	0.0391 (2)	
Cl3	0.24483 (7)	0.27221 (2)	0.99017 (10)	0.0428 (2)	
C37	0.2369 (2)	0.31996 (9)	0.8865 (3)	0.0328 (7)	
H37	0.3077	0.3322	0.8923	0.039*	
Cl4	1.05290 (6)	0.31392 (3)	0.32391 (10)	0.0456 (2)	
Cl5	1.01463 (7)	0.39768 (3)	0.20247 (12)	0.0556 (3)	
Cl6	0.86232 (7)	0.33276 (3)	0.12830 (11)	0.0590 (3)	
C38	0.9583 (2)	0.35249 (10)	0.2702 (4)	0.0385 (8)	
H38	0.9263	0.3602	0.3632	0.046*	

### Atomic displacement parameters (Å^2^)

**Table:**

	*U*^11^	*U*^22^	*U*^33^	*U*^12^	*U*^13^	*U*^23^
S1	0.0279 (4)	0.0193 (3)	0.0321 (4)	0.0014 (3)	0.0086 (3)	0.0029 (3)
O1	0.0336 (12)	0.0225 (10)	0.0444 (14)	0.0006 (9)	0.0154 (10)	0.0023 (10)
O2	0.0273 (11)	0.0208 (10)	0.0294 (11)	−0.0001 (8)	0.0077 (9)	−0.0004 (9)
O3	0.0362 (12)	0.0253 (11)	0.0439 (13)	0.0022 (9)	0.0185 (11)	0.0012 (10)
N1	0.0263 (13)	0.0197 (12)	0.0277 (14)	−0.0027 (10)	0.0080 (11)	0.0030 (10)
N2	0.0251 (13)	0.0217 (12)	0.0302 (14)	−0.0007 (10)	0.0061 (11)	0.0017 (11)
N3	0.0296 (13)	0.0216 (12)	0.0289 (14)	−0.0014 (10)	0.0063 (11)	0.0000 (11)
N4	0.0273 (13)	0.0202 (12)	0.0323 (14)	0.0012 (10)	0.0119 (11)	0.0009 (11)
C1	0.0267 (16)	0.0230 (15)	0.0258 (17)	−0.0009 (12)	0.0072 (13)	−0.0029 (13)
C2	0.0248 (15)	0.0216 (14)	0.0244 (16)	0.0005 (12)	0.0018 (13)	−0.0023 (12)
C3	0.0285 (15)	0.0196 (14)	0.0206 (16)	−0.0015 (12)	0.0035 (13)	−0.0002 (12)
C4	0.0254 (15)	0.0198 (14)	0.0232 (16)	−0.0017 (12)	0.0032 (13)	−0.0005 (12)
C5	0.0242 (15)	0.0218 (15)	0.0299 (17)	0.0012 (13)	0.0047 (13)	0.0042 (13)
C6	0.0230 (15)	0.0290 (16)	0.0264 (17)	0.0030 (13)	0.0050 (13)	−0.0007 (13)
C7	0.0236 (15)	0.0247 (15)	0.0246 (16)	−0.0029 (12)	0.0019 (13)	0.0002 (13)
C8	0.0282 (16)	0.0231 (15)	0.0351 (18)	−0.0020 (13)	0.0070 (14)	−0.0052 (14)
C9	0.0304 (17)	0.0334 (17)	0.0363 (19)	0.0006 (14)	0.0130 (15)	−0.0042 (15)
C10	0.0318 (17)	0.0328 (17)	0.040 (2)	−0.0058 (14)	0.0138 (15)	0.0029 (15)
C11	0.0292 (16)	0.0262 (16)	0.0316 (17)	0.0009 (13)	0.0100 (14)	0.0039 (14)
C12	0.0304 (17)	0.0242 (16)	0.0306 (17)	0.0021 (13)	0.0096 (14)	−0.0007 (13)
C13	0.0314 (16)	0.0232 (15)	0.0266 (17)	0.0017 (13)	0.0068 (14)	−0.0022 (13)
C14	0.0396 (18)	0.0210 (15)	0.0353 (18)	−0.0008 (13)	0.0107 (15)	−0.0047 (14)
C15	0.0354 (17)	0.0223 (15)	0.0334 (18)	−0.0041 (13)	0.0090 (15)	0.0008 (14)
C16	0.0292 (16)	0.0215 (15)	0.0253 (16)	−0.0055 (13)	0.0031 (13)	0.0016 (13)
C17	0.0267 (16)	0.0255 (16)	0.0292 (17)	−0.0023 (13)	0.0046 (14)	0.0022 (13)
C18	0.0241 (15)	0.0258 (16)	0.0259 (16)	−0.0011 (12)	0.0030 (13)	0.0016 (13)
C19	0.0266 (16)	0.0251 (15)	0.0289 (17)	−0.0030 (13)	0.0038 (14)	0.0007 (13)
C20	0.0292 (16)	0.0251 (15)	0.0351 (18)	0.0008 (13)	0.0095 (14)	0.0008 (14)
C21	0.0356 (18)	0.0314 (17)	0.041 (2)	0.0021 (14)	0.0182 (16)	0.0017 (15)
C22	0.0310 (17)	0.0286 (16)	0.045 (2)	−0.0043 (14)	0.0158 (16)	0.0068 (15)
C23	0.0343 (17)	0.0231 (15)	0.0396 (19)	−0.0022 (13)	0.0088 (15)	0.0018 (14)
C24	0.0305 (16)	0.0213 (14)	0.0264 (17)	−0.0014 (13)	0.0048 (14)	0.0014 (13)
C25	0.0253 (16)	0.0192 (15)	0.0355 (18)	−0.0024 (12)	0.0132 (14)	0.0044 (13)
C26	0.0327 (18)	0.0286 (17)	0.047 (2)	0.0010 (14)	0.0113 (16)	−0.0046 (15)
C27	0.046 (2)	0.0222 (16)	0.068 (3)	0.0012 (15)	0.025 (2)	−0.0064 (17)
C28	0.042 (2)	0.0302 (18)	0.069 (3)	0.0100 (16)	0.027 (2)	0.0088 (19)
C29	0.0312 (18)	0.049 (2)	0.060 (3)	0.0069 (16)	0.0106 (18)	0.010 (2)
C30	0.0305 (17)	0.0358 (18)	0.040 (2)	−0.0005 (14)	0.0053 (15)	0.0002 (15)
C31	0.0262 (15)	0.0176 (14)	0.0276 (17)	0.0026 (12)	0.0103 (13)	−0.0005 (13)
C32	0.0274 (16)	0.0243 (15)	0.0310 (18)	0.0001 (13)	0.0064 (14)	−0.0031 (14)
C33	0.0365 (18)	0.0275 (16)	0.0358 (19)	0.0063 (14)	0.0080 (15)	0.0059 (14)
C34	0.0403 (19)	0.0204 (15)	0.041 (2)	0.0021 (14)	0.0141 (17)	0.0074 (14)
C35	0.0352 (17)	0.0224 (15)	0.0375 (19)	−0.0069 (13)	0.0111 (15)	−0.0031 (14)
C36	0.0270 (15)	0.0221 (15)	0.0256 (16)	−0.0016 (13)	0.0028 (13)	0.0013 (13)
Cl1	0.0452 (5)	0.0326 (4)	0.0310 (4)	0.0049 (4)	0.0039 (4)	−0.0022 (4)
Cl2	0.0468 (5)	0.0352 (4)	0.0373 (5)	0.0007 (4)	0.0129 (4)	−0.0049 (4)
Cl3	0.0531 (5)	0.0313 (4)	0.0430 (5)	−0.0014 (4)	0.0043 (4)	0.0102 (4)
C37	0.0353 (17)	0.0287 (17)	0.0343 (19)	−0.0015 (14)	0.0049 (15)	0.0005 (14)
Cl4	0.0384 (5)	0.0557 (5)	0.0414 (5)	0.0030 (4)	0.0017 (4)	0.0052 (4)
Cl5	0.0587 (6)	0.0352 (5)	0.0798 (7)	0.0018 (4)	0.0321 (5)	−0.0095 (5)
Cl6	0.0527 (6)	0.0570 (6)	0.0582 (6)	−0.0022 (5)	−0.0200 (5)	−0.0019 (5)
C38	0.0342 (18)	0.045 (2)	0.037 (2)	−0.0028 (15)	0.0050 (15)	−0.0029 (16)

### Geometric parameters (Å, °)

**Table:**

S1—C4	1.721 (3)	C17—H17	0.9500
S1—C1	1.721 (3)	C18—C23	1.401 (4)
O1—C5	1.221 (3)	C18—C19	1.412 (4)
O2—C13	1.361 (3)	C19—C20	1.384 (4)
O2—C16	1.374 (3)	C20—C21	1.378 (4)
O3—C24	1.221 (3)	C20—H20	0.9500
N1—C5	1.362 (3)	C21—C22	1.385 (4)
N1—C6	1.409 (3)	C21—H21	0.9500
N1—H1N	0.8999	C22—C23	1.379 (4)
N2—C12	1.282 (3)	C22—H22	0.9500
N2—C7	1.418 (3)	C23—H23	0.9500
N3—C17	1.276 (3)	C25—C26	1.373 (4)
N3—C18	1.411 (3)	C25—C30	1.385 (4)
N4—C24	1.354 (3)	C26—C27	1.399 (4)
N4—C19	1.410 (3)	C26—H26	0.9500
N4—H4N	0.8999	C27—C28	1.369 (5)
C1—C2	1.377 (4)	C27—H27	0.9500
C1—C24	1.486 (4)	C28—C29	1.369 (5)
C2—C3	1.427 (4)	C28—H28	0.9500
C2—C25	1.495 (4)	C29—C30	1.369 (4)
C3—C4	1.380 (4)	C29—H29	0.9500
C3—C31	1.493 (4)	C30—H30	0.9500
C4—C5	1.497 (4)	C31—C32	1.383 (4)
C6—C11	1.393 (4)	C31—C36	1.387 (4)
C6—C7	1.412 (4)	C32—C33	1.392 (4)
C7—C8	1.391 (4)	C32—H32	0.9500
C8—C9	1.377 (4)	C33—C34	1.368 (4)
C8—H8	0.9500	C33—H33	0.9500
C9—C10	1.378 (4)	C34—C35	1.377 (4)
C9—H9A	0.9500	C34—H34	0.9500
C10—C11	1.385 (4)	C35—C36	1.393 (4)
C10—H10	0.9500	C35—H35	0.9500
C11—H11	0.9500	C36—H36	0.9500
C12—C13	1.431 (4)	Cl1—C37	1.768 (3)
C12—H12	0.9500	Cl2—C37	1.748 (3)
C13—C14	1.368 (4)	Cl3—C37	1.765 (3)
C14—C15	1.402 (4)	C37—H37	1.0000
C14—H14	0.9500	Cl4—C38	1.757 (3)
C15—C16	1.357 (4)	Cl5—C38	1.761 (3)
C15—H15	0.9500	Cl6—C38	1.741 (3)
C16—C17	1.442 (4)	C38—H38	1.0000
			
C4—S1—C1	92.08 (13)	C20—C19—C18	120.6 (3)
C13—O2—C16	106.2 (2)	N4—C19—C18	115.4 (2)
C5—N1—C6	129.4 (2)	C21—C20—C19	119.9 (3)
C5—N1—H1N	118.3	C21—C20—H20	120.1
C6—N1—H1N	112.3	C19—C20—H20	120.1
C12—N2—C7	120.6 (2)	C20—C21—C22	120.4 (3)
C17—N3—C18	120.9 (2)	C20—C21—H21	119.8
C24—N4—C19	129.0 (2)	C22—C21—H21	119.8
C24—N4—H4N	118.4	C23—C22—C21	120.4 (3)
C19—N4—H4N	112.4	C23—C22—H22	119.8
C2—C1—C24	127.0 (2)	C21—C22—H22	119.8
C2—C1—S1	111.4 (2)	C22—C23—C18	120.4 (3)
C24—C1—S1	121.5 (2)	C22—C23—H23	119.8
C1—C2—C3	112.7 (2)	C18—C23—H23	119.8
C1—C2—C25	123.8 (2)	O3—C24—N4	124.9 (3)
C3—C2—C25	123.5 (2)	O3—C24—C1	121.4 (2)
C4—C3—C2	112.1 (2)	N4—C24—C1	113.7 (2)
C4—C3—C31	125.8 (2)	C26—C25—C30	118.5 (3)
C2—C3—C31	122.1 (2)	C26—C25—C2	121.8 (3)
C3—C4—C5	127.2 (2)	C30—C25—C2	119.7 (3)
C3—C4—S1	111.7 (2)	C25—C26—C27	120.6 (3)
C5—C4—S1	120.81 (19)	C25—C26—H26	119.7
O1—C5—N1	124.5 (3)	C27—C26—H26	119.7
O1—C5—C4	121.7 (2)	C28—C27—C26	119.8 (3)
N1—C5—C4	113.7 (2)	C28—C27—H27	120.1
C11—C6—N1	123.9 (2)	C26—C27—H27	120.1
C11—C6—C7	120.2 (3)	C29—C28—C27	119.7 (3)
N1—C6—C7	115.9 (2)	C29—C28—H28	120.1
C8—C7—C6	118.6 (3)	C27—C28—H28	120.1
C8—C7—N2	126.6 (2)	C30—C29—C28	120.6 (3)
C6—C7—N2	114.8 (2)	C30—C29—H29	119.7
C9—C8—C7	120.9 (3)	C28—C29—H29	119.7
C9—C8—H8	119.5	C29—C30—C25	120.9 (3)
C7—C8—H8	119.5	C29—C30—H30	119.6
C8—C9—C10	119.9 (3)	C25—C30—H30	119.6
C8—C9—H9A	120.0	C32—C31—C36	119.1 (2)
C10—C9—H9A	120.0	C32—C31—C3	119.5 (2)
C9—C10—C11	121.1 (3)	C36—C31—C3	121.2 (3)
C9—C10—H10	119.5	C31—C32—C33	120.3 (3)
C11—C10—H10	119.5	C31—C32—H32	119.8
C10—C11—C6	119.2 (3)	C33—C32—H32	119.8
C10—C11—H11	120.4	C34—C33—C32	120.4 (3)
C6—C11—H11	120.4	C34—C33—H33	119.8
N2—C12—C13	124.1 (3)	C32—C33—H33	119.8
N2—C12—H12	118.0	C33—C34—C35	119.7 (3)
C13—C12—H12	118.0	C33—C34—H34	120.2
O2—C13—C14	110.1 (2)	C35—C34—H34	120.2
O2—C13—C12	120.1 (2)	C34—C35—C36	120.5 (3)
C14—C13—C12	129.8 (3)	C34—C35—H35	119.8
C13—C14—C15	106.7 (2)	C36—C35—H35	119.8
C13—C14—H14	126.6	C31—C36—C35	119.9 (3)
C15—C14—H14	126.6	C31—C36—H36	120.0
C16—C15—C14	106.8 (2)	C35—C36—H36	120.0
C16—C15—H15	126.6	Cl2—C37—Cl3	109.78 (16)
C14—C15—H15	126.6	Cl2—C37—Cl1	110.25 (16)
C15—C16—O2	110.1 (2)	Cl3—C37—Cl1	109.00 (16)
C15—C16—C17	129.9 (3)	Cl2—C37—H37	109.3
O2—C16—C17	120.0 (2)	Cl3—C37—H37	109.3
N3—C17—C16	123.3 (3)	Cl1—C37—H37	109.3
N3—C17—H17	118.3	Cl6—C38—Cl4	109.85 (17)
C16—C17—H17	118.3	Cl6—C38—Cl5	110.45 (18)
C23—C18—N3	126.4 (3)	Cl4—C38—Cl5	110.26 (17)
C23—C18—C19	118.3 (3)	Cl6—C38—H38	108.7
N3—C18—C19	115.3 (2)	Cl4—C38—H38	108.7
C20—C19—N4	124.0 (3)	Cl5—C38—H38	108.7
			
C4—S1—C1—C2	0.3 (2)	C18—N3—C17—C16	−179.5 (3)
C4—S1—C1—C24	−178.1 (2)	C15—C16—C17—N3	176.6 (3)
C24—C1—C2—C3	178.4 (3)	O2—C16—C17—N3	−3.3 (4)
S1—C1—C2—C3	0.1 (3)	C17—N3—C18—C23	−0.9 (4)
C24—C1—C2—C25	1.0 (5)	C17—N3—C18—C19	179.5 (3)
S1—C1—C2—C25	−177.3 (2)	C24—N4—C19—C20	−1.4 (5)
C1—C2—C3—C4	−0.6 (3)	C24—N4—C19—C18	179.5 (3)
C25—C2—C3—C4	176.8 (3)	C23—C18—C19—C20	−0.4 (4)
C1—C2—C3—C31	−178.7 (3)	N3—C18—C19—C20	179.2 (3)
C25—C2—C3—C31	−1.4 (4)	C23—C18—C19—N4	178.7 (3)
C2—C3—C4—C5	−172.8 (3)	N3—C18—C19—N4	−1.7 (4)
C31—C3—C4—C5	5.3 (5)	N4—C19—C20—C21	−178.8 (3)
C2—C3—C4—S1	0.8 (3)	C18—C19—C20—C21	0.2 (4)
C31—C3—C4—S1	178.9 (2)	C19—C20—C21—C22	0.2 (5)
C1—S1—C4—C3	−0.7 (2)	C20—C21—C22—C23	−0.4 (5)
C1—S1—C4—C5	173.4 (2)	C21—C22—C23—C18	0.2 (5)
C6—N1—C5—O1	1.3 (5)	N3—C18—C23—C22	−179.3 (3)
C6—N1—C5—C4	−177.0 (3)	C19—C18—C23—C22	0.2 (4)
C3—C4—C5—O1	−24.3 (4)	C19—N4—C24—O3	−0.9 (5)
S1—C4—C5—O1	162.6 (2)	C19—N4—C24—C1	178.9 (3)
C3—C4—C5—N1	154.1 (3)	C2—C1—C24—O3	24.1 (5)
S1—C4—C5—N1	−19.0 (3)	S1—C1—C24—O3	−157.8 (2)
C5—N1—C6—C11	4.6 (5)	C2—C1—C24—N4	−155.7 (3)
C5—N1—C6—C7	−175.9 (3)	S1—C1—C24—N4	22.5 (3)
C11—C6—C7—C8	−1.2 (4)	C1—C2—C25—C26	−95.4 (4)
N1—C6—C7—C8	179.3 (2)	C3—C2—C25—C26	87.5 (4)
C11—C6—C7—N2	176.6 (2)	C1—C2—C25—C30	85.3 (4)
N1—C6—C7—N2	−2.9 (4)	C3—C2—C25—C30	−91.8 (3)
C12—N2—C7—C8	5.3 (4)	C30—C25—C26—C27	0.4 (4)
C12—N2—C7—C6	−172.3 (3)	C2—C25—C26—C27	−178.9 (3)
C6—C7—C8—C9	1.5 (4)	C25—C26—C27—C28	0.3 (5)
N2—C7—C8—C9	−175.9 (3)	C26—C27—C28—C29	−0.7 (5)
C7—C8—C9—C10	0.0 (5)	C27—C28—C29—C30	0.5 (5)
C8—C9—C10—C11	−2.0 (5)	C28—C29—C30—C25	0.1 (5)
C9—C10—C11—C6	2.3 (5)	C26—C25—C30—C29	−0.6 (4)
N1—C6—C11—C10	178.7 (3)	C2—C25—C30—C29	178.8 (3)
C7—C6—C11—C10	−0.7 (4)	C4—C3—C31—C32	−110.0 (3)
C7—N2—C12—C13	177.5 (3)	C2—C3—C31—C32	67.9 (4)
C16—O2—C13—C14	0.3 (3)	C4—C3—C31—C36	73.7 (4)
C16—O2—C13—C12	−178.4 (3)	C2—C3—C31—C36	−108.4 (3)
N2—C12—C13—O2	−0.4 (5)	C36—C31—C32—C33	−1.0 (4)
N2—C12—C13—C14	−178.9 (3)	C3—C31—C32—C33	−177.4 (3)
O2—C13—C14—C15	0.1 (3)	C31—C32—C33—C34	2.0 (4)
C12—C13—C14—C15	178.7 (3)	C32—C33—C34—C35	−1.3 (4)
C13—C14—C15—C16	−0.4 (3)	C33—C34—C35—C36	−0.4 (4)
C14—C15—C16—O2	0.7 (3)	C32—C31—C36—C35	−0.6 (4)
C14—C15—C16—C17	−179.2 (3)	C3—C31—C36—C35	175.7 (3)
C13—O2—C16—C15	−0.6 (3)	C34—C35—C36—C31	1.3 (4)
C13—O2—C16—C17	179.3 (2)		

### Hydrogen-bond geometry (Å, °)

**Table:**

*D*—H···*A*	*D*—H	H···*A*	*D*···*A*	*D*—H···*A*
N1—H1N···N2	0.90	2.13	2.622 (3)	114
N1—H1N···S1	0.90	2.48	2.970 (3)	115
N4—H4N···N3	0.90	2.13	2.620 (3)	114
N4—H4N···S1	0.90	2.53	2.986 (3)	112
C32—H32···Cl6^i^	0.95	2.69	3.461 (3)	139
C37—H37···O1	1.00	2.34	3.149 (3)	137
C38—H38···O3	1.00	2.46	3.205 (3)	131

Symmetry codes: (i) *x*, *y*, *z*+1.
